# The role of endoscopic ultrasound in children with Pancreatobiliary and gastrointestinal disorders: a single center series and review of the literature

**DOI:** 10.1186/s12887-017-0956-z

**Published:** 2017-12-06

**Authors:** Alessandro Fugazza, Barbara Bizzarri, Federica Gaiani, Marco Manfredi, Alessia Ghiselli, Pellegrino Crafa, Maria Clotilde Carra, Nicola de’Angelis, Gian Luigi de’Angelis

**Affiliations:** 1 Gastroenterology and Endoscopy Unit, University Hospital of Parma, Via Gramsci 14, 43126 Parma, Italy; 2 Department of Pediatrics, “Pietro Barilla” Children’s Hospital, University Hospital of Parma, Via Gramsci 14, 43126 Parma, Italy; 3 Department of Pathology, University Hospital of Parma, 43126 Parma, Italy; 4 University Paris VII, Rothschild Hospital, AP-HP, Paris, France; 5 Unit of Digestive, Hepato-Pancreato-Biliary Surgery and Liver Transplantation, Henri Mondor Hospital, AP-HP, 94010 Paris, Créteil France; 6 Cancer Research Lab. EC2M3, Université Paris-Est, Créteil, Val de Marne UPEC, 94010 Paris, France

**Keywords:** Endoscopic ultrasound, Gastrointestinal disease, Pancreatobiliary disease, Pediatrics

## Abstract

**Abstract:**

**Background:**

The role of endoscopic ultrasound (EUS) in the management of pancreatobiliary and digestive diseases is well established in adults, but it remains limited in children. The aim of this study was to evaluate the feasibility, safety, and clinical impact of EUS use in children.

**Methods:**

This is a retrospective analysis of a prospectively acquired database of consecutive pediatric (< 18 years) patients presenting an indication for EUS for pancreatobiliary and gastrointestinal disorders.

**Results:**

Between January 2010 and January 2016, 47 procedures were performed in 40 children (mean age of 15.1 ± 4.7 years; range 3–18). The majority of EUS (*n* = 32; 68.1%) were performed for pancreatobiliary and upper gastrointestinal pathologies, including suspected common bile duct stones (CBDs), acute biliary pancreatitis, recurrent/chronic pancreatitis, cystic pancreatic mass, recurrent hypoglycemia, duodenal polyp, gastric submucosal lesion, and perigastric abscess. In only 2 out of 18 children with suspected CBDs or acute biliary pancreatitis, EUS confirmed CBDs. EUS-guided fine needle aspiration was performed in 3 (6.4%) patients. Fifteen (31.9%) procedures were performed for lower gastrointestinal tract disorders, including suspected anal Crohn’s disease, fecal incontinence, and encopresis. Overall, EUS had a significant impact on the subsequent clinical management in 87.2% of patients.

**Conclusion:**

The present findings were consistent with results observed in the current relevant literature and support EUS as a safe and feasible diagnostic and therapeutic tool, which yields a significant clinical impact in children with pancreatobiliary and gastrointestinal disorders.

## Background

Endoscopic ultrasound (EUS) and EUS-guided fine needle aspiration (FNA) have been dramatically evolving since their introduction and has become one of the most important techniques for the definitive cytological or histological diagnosis and the management of pancreatobiliary and gastrointestinal (GI) diseases [1–3].

Historically, the primary technique used for the diagnosis and treatment of several pancreatic and biliary diseases in both adults and children was endoscopic retrograde cholangiopancreatography (ERCP) [4, 5]. Recent studies performed in adult populations have identified computed tomography (CT), magnetic resonance cholangiopancreatography (MRCP) and EUS as non-invasive tests that can be used as an alternative to ERCP for pancreatobiliary diseases [6–8] to minimize the risk of associated complications and to eventually prevent unnecessary and invasive diagnostic procedures [4]. In particular, MRCP and EUS are radiation-free imaging exams that are now considered as the best methods for the detection of common bile duct stones (CBDs), yielding the highest diagnostic accuracy [9, 10].

While the role of EUS in adults is well established and widespread, EUS and EUS-FNA in children are supported by limited number of studies, and its indications are restricted compared to adults [1, 3, 8, 11–17]. This may be due to multiple factors, including the low incidence of pancreatobiliary disorders and GI tumors in the pediatric population [12], an insufficient awareness among pediatricians, and the limited experience of pediatric endoscopists. Indeed, most EUS procedures in children are performed by adult gastroenterologists because the low number of pediatric EUS procedures does not enable pediatric gastroenterologists to acquire and maintain proficiency in EUS [12, 13]. However, EUS may have an important clinical impact in children, and efforts should be made to disperse this technique as a valuable diagnostic and therapeutic tool, which minimizes the procedural risks and avoids unnecessary ERCP [1, 15].

The present study aims to report the experience of a single high-volume gastroenterology and endoscopy unit in the application of EUS and EUS-FNA in children to further evaluate its feasibility, safety, and clinical impact on pediatric pancreatobiliary and GI disorders. In addition, the present findings are discussed in comparison with current pertinent literature.

## Methods

### Study population

The present study is a retrospective analysis of a prospectively acquired database of consecutive pediatric (< 18 years) patients presenting an indication for EUS or EUS-FNA. All procedures were performed between January 2010 and January 2016 at the Endoscopy Unit of the University of Parma. EUS and EUS-FNA were performed by a senior gastroenterologist (GLdeA) with expertise in both adult and pediatric endoscopy.

Written consent was obtained from both parents or legal guardians, and it included consent for the therapeutic procedures. All data were collected in compliance with the ethical principles stated in the Declaration of Helsinki, and according to the Good Clinical Practice protocols and Privacy Protection Law of the institution.

### Techniques

Upper EUS examinations were performed with patients under deep sedation or general anesthesia performed by a pediatric anesthesiologist depending on the American Society of Anesthesiologists (ASA) classification and the type of procedure. Lower EUS were generally performed without sedation unless specific conditions (e.g., very young age) contraindicated it. A minimum of 10 to 12 h of fasting were required for upper EUS, whereas 2 enemas were requested before lower EUS.

EUS procedures were performed using different echoendoscopes, including radial echoendoscopes (insertion tube of 13.45 mm, biopsy channel of 2.4 mm; Pentax EG-3670URK, Pentax Hamburg, Germany); linear echoendoscopes (insertion tube of 12.8 mm, biopsy channel of 3.8 mm; Pentax EG-3870UTK, Pentax Hamburg, Germany); linear Slim echoendoscopes (insertion tube of 10.8 mm, biopsy channel 2.8 mm; Pentax EG-3270UK, Pentax Hamburg, Germany); or linear ultrasound bronchoscope (insertion tube of 6.3 mm, biopsy channel of 2 mm; Pentax EB1970UK, Pentax Hamburg, Germany) with a Hitachi – Aloka Avius processor (Hitachi, Hamburg, Germany).

The choice of the scope was based on the age and weight of the patient. Specifically, the linear Slim echoendoscope was used for upper echoendoscopy in children younger than 10 years and/or weighing less than 35 kg (cases 5, 15, 20 and 32; Table 2), while the linear ultrasound bronchoscope was chosen only for the management of case 33, as the child was 4 years old and weighed 13 kg.

Examination of the pancreatic head, biliary tract, gallbladder, and portal regions was performed from the descending duodenum and duodenal bulb; the pancreatic body and tail, and the left lobe of the liver were visualized from the stomach. For lower EUS, the instrument was advanced beyond the rectum, and imaging was performed on slow scope withdrawal after instilling water into the rectum to examine the rectosigmoid junction, rectum and anal canal [14].

EUS-FNA was performed using either a 22- or 25-gauge FNA biopsy needle (EchoTip, Wilson-Cook Medical Inc., Winston-Salem, NC) with color Doppler imaging to exclude vessels along the path of the needle. To increase diagnostic accuracy, two or three needle passes were made for solid lesions. Elastography, an indicator of tissue stiffness, was used for differential diagnosis and to address the sampling of solid lesions. One pass was performed for cystic lesions to minimize infection complications. Intravenous antibiotic prophylaxis was administered before EUS-FNA of cystic lesions. Drainage of pancreatic pseudocyst was performed in the most prominent site of the bulge using a 19-gauge needle (EchoTip, Wilson-Cook Medical Inc., Winston-Salem, NC). A 0.035-in. guidewire (Microvasive Endoscopy, Boston Scientific Corp, Galway, Ireland) was inserted through the needle into the pseudocyst under X-ray control. After removal of the needle, a cyst-gastrostome was inserted. Finally, the gastric wall was dilated up to 10 mm using a wire-guided balloon and a flared-type biflanged metal stent (30 mm length, 10.5 Fr, Niti-S Nagi stent, Taewoong Medical Co., Seoul, Korea) was inserted into the cyst cavity.

All adverse events, defined as any event that negatively impacted on the health status of the patient within 30 days from the procedure, were observed via outpatient assessments for the first 2 weeks and by weekly telephone contacts with family members and/or referring physicians afterwards.

### Study outcomes

Patient demographics, relevant medical history, initial diagnosis, previous conventional abdominal imaging (ultrasound (US), CT, magnetic resonance imaging (MRI) or MRCP), indication for EUS, specific EUS findings, therapeutic interventions, impact of EUS on the patient’s subsequent management, and complications were reviewed and analyzed.

The clinical impact of EUS was scored as [16]:

(0) No impact on diagnosis or management;

(1) Establishment of a definitive diagnosis or exclusion of suspected pathological conditions;

(2) Yield of new, relevant findings, which subsequently altered the patient management strategy.

(3) Yield of relevant findings and EUS-based therapeutic approach.

### Pathological examination

After FNA, the aspirated material was first smeared on a glass slide by the operating endoscopist, taking care that any clotted material was preserved for a cell block. In this case, the material was placed into a container of 10% neutral-buffered formalin fixative for the creation of a tissue block. Air-dried (for Diff-Quick staining) and fixed smears (fixed immediately in 95% ethyl alcohol for subsequent Papanicolaou staining) were prepared in an almost equal ratio. All slides were analyzed by an experienced cytopathologist (PC).

### Statistical analysis

Data are reported as the mean and standard deviation or range for continuous variables and as relative frequencies (number and percentages) for categorical variables. The outcome measures (mean values, standard deviation, and ranges) were extracted from the original relevant articles in the analysis of the current literature. Whenever possible, the overall data were analyzed as the sum or weighted mean (and standard deviation).

## Results

During the study period, a total of 2161 EUS were performed in the unit, of which 47 (2.17%) pediatric EUS procedures in 40 patients (18 females, 22 males; mean age of 15.1 ± 4.7 years, range 3–18). These included 32 (68.1%) upper EUS and 15 (31.9%) lower EUS (Table 1). All EUS procedures were performed in the Endoscopy Unit (not operating room).

**Table 1:** Study population and indications for EUS

Children/Procedures [*n*]	40/47
Females/Males [*n*]	18/22
Age [y, range]	3–18
[y, mean ± SD]	15.1 ± 4.7
Indications for EUS [*n* (%)]	
Upper-GI EUS	32 (68.1)
• Suspected CBDs	8
• Acute biliary pancreatitis	7
• Recurrent/chronic pancreatitis	4
• Suspected CBDs in patients with UC	3
• Cystic pancreatic mass	3
• Recurrent hypoglycemia	2
• Duodenal polyp	2
• Pseudocyst drainage	1
• Gastric submucosal lesion	1
• Perigastric abscess	1
Lower-GI EUS	15 (31.9)
• Suspected anal Crohn’s Disease	12
• Fecal incontinence	2
• Encopresis	1
EUS-FNA [n (%)]	3 (6.4)
EUS procedures with sedation [n (%)]	
○Upper GI	
• Deep sedation	22 (46.8)
• General anesthesia	10 (21.3)
○Lower GI	
• No sedation	14 (29.8)
• Deep sedation	1 (2.1)
Anesthesia-related adverse events [n (%)]	0
Clinical Impact of EUS [n (%)]	
• Score 0	6 (12.8)
• Score 1	24 (51)
• Score 2	17 (36.2)
Significant impact (score 1 + 2)	41 (87.2)

*EUS* indicates endoscopic ultrasound; *CBDs* indicates common bile ducts stones; *UC* indicates ulcerative colitis; *FNA* indicates fine needle aspiration; *GI* indicates gastrointestinal

The majority of EUS investigated the pancreatobiliary tract (59.5%), followed by the rectum (31.9%), stomach (4.3%), and duodenum (4.3%). Overall, 3 (6.4%) EUS-FNA were performed with a diagnostic yield of 100%. All 47 procedures were technically successful, and no adverse events, intraoperative or delayed complications occurred. Details of the EUS indications and findings are described below by the organs involved and are shown in Table 2.

**Table 2:** EUS procedures by indications and findings

Case	Age (y) / Sex	Indication	Comorbidities	Imagery/Diagnostic Studies Prior EUS	Sedation	EUS Findings	Treatment	Impact
1	18 F	Suspected CBDs	Nil	US, CT	DP	Gallstones and CBDs	Stones extracted at ERCP; laparoscopic cholecystectomy	2
2	12 F	Suspected acute biliary pancreatitis	Psoriasis	US, CT	DP	Normal	Precluded need for ERCP	1
3	18 M	Suspected CBDs	Ulcerative colitis, sclerosing cholangitis	US, MRI	DP	Normal	Precluded need for ERCP	1
4	12 F	Suspected CBDs	Nil	US	DP	Gallstones	Precluded need for ERCP; laparoscopic cholecystectomy	1
5	7 M	Recurrent pancreatitis	Klinefelter syndrome	US, MRI	GA	Chronic pancreatitis	Nil	1
5b		Recurrent pancreatitis (1 year later)	Nil	CT	GA	Chronic pancreatitis	Nil	0
6	14 M	Acute biliary pancreatitis	Nil	US, CT	DP	Gallstones, edematous pancreatitis	Precluded need for ERCP; laparoscopic cholecystectomy	1
7	15 M	Suspected CBDs	Nil	US	DP	Gallstones	Precluded need for ERCP; Laparoscopic cholecystectomy	1
8	18 M	Recurrent hypoglycemia	Nil	CT, MRI	DP	Solid hypoechogenic hypervascular lesion of pancreatic tail	FNA with 25 G, diagnosis of insulinomas; surgical resection	2
9	18 M	Suspected anal Crohn’s disease	Rectal Crohn’s disease	Colonoscopy, MRI	NS	Normal	Nil	0
10	18 M	Suspected CBDs	Nil	US	DP	Gallstones	Precluded need for ERCP; laparoscopic cholecystectomy	1
11	18 M	Suspected anal Crohn’s disease	Rectal Crohn’s disease	Colonoscopy, CT	NS	Trans-sphincteric fistula	Biologic therapy	2
11b		Control after 6 months of therapy	Nil	EUS	NS	Partial remission	Biologic therapy	2
11c		Control after 1 year of therapy	Nil	EUS	NS	Remission	Stop of biologic therapy	2
12	18 F	Suspected CBDs	Nil	US, MRI	DP	Gallstones	Precluded need for ERCP; laparoscopic cholecystectomy	1
13	16 M	Suspected Crohn’s disease	Rectal Crohn’s disease	Colonoscopy, CT	NS	Normal	Nil	0
14	13 F	Recurrent pancreatitis	Celiac disease	US, CT	DP	Chronic pancreatitis	Nil	1
15	9 M	Suspected acute biliary pancreatitis	Nil	US, CT	DP	Gallstones edematous pancreatitis	Precluded need for ERCP; laparoscopic cholecystectomy	1
16	16 F	Cystic pancreatic mass	Takayasu arteritis Hashimoto thyroiditis	US	GA	Voluminous head pancreatic cysts	FNA with 22 G (serous cystadenoma)	1
16b		Acute pancreatitis	Nil	CT	GA	Compression of CBD and Wirsung duct	Whipple resection	2
17	12 M	Fecal incontinence	Surgery for Hirschsprung disease	MRI	NS	Interruption of internal anal sphincter	Symptomatic management	1
18	18 F	Suspected anal Crohn’s disease	Ileo-colonic Crohn’s disease	Colonoscopy, CT	NS	Normal	Nil	0
19	12 M	Suspected anal Crohn’s disease	Colonic Crohn’s disease	Colonoscopy	NS	Extra sphincteric fistula	Biologic therapy	2
19b		Control after 6 months of therapy	Nil	EUS	NS	Remission	Biologic therapy	2
20	9 F	Duodenal polyp	Nil	EGD, MRI, PET with Ga-DOTATOC	GA	Hypoechoic, hypervascular lesion originate in the III layer, infiltrate the IV	Surgical resection (NET G2)	2
20b		Follow up after surgery	Nil	MRI, CT, PET with Ga-DOTATOC	GA	Normal	Nil	0
21	13 F	Suspected acute biliary pancreatitis	Nil	US, MRI	DP	Gallstones, acute necrotizing pancreatitis	Precluded need for ERCP; laparoscopic cholecystectomy	1
21b		Abdominal pain	Nil	CT	GA	Pancreatic pseudocyst	Transgastric drainage with metallic stent	2
22	12 F	Fecal incontinence	Surgery for Hirschsprung disease	MRI	NS	Interruption of internal anal sphincter	Symptomatic management	1
23	15 M	Suspected CBDs	Ulcerative colitis sclerosing cholangitis	US, MRI	DP	Normal	Precluded need for ERCP	1
24	17 F	Suspected anal Crohn’s disease	Colonic Crohn’s disease	Colonoscopy, MRI	NS	Abscess with extra sphincteric fistula	Surgical intervention	2
25	18 M	Suspected CBDs	Ulcerative Colitis sclerosing cholangitis	US, MRI	DP	Normal	Precluded need for ERCP	1
26	17 M	Suspected CBDs	Nil	US	DP	Gallstones	Precluded need for ERCP; laparoscopic cholecystectomy	1
27	18 M	Suspected Crohn’s anal disease	Ileo-colonic Crohn’s disease	Colonoscopy, CT	NS	Abscess with extra sphincteric fistula	Surgical intervention	2
28	12 M	Recurrent pancreatitis	Nil	MRI	DP	Chronic pancreatitis	Nil	1
29	17 M	Suspected acute biliary pancreatitis	Nil	US, CT	DP	Gallstones, edematous pancreatitis	Precluded need for ERCP; laparoscopic cholecystectomy	1
30	18 M	Suspected Crohn’s anal disease	Nil	Colonoscopy, MRI	NS	Abscess with extra sphincteric fistula	Surgical intervention	2
31	14 F	Gastric subepithelial lesions	Nil	EGD	DP	Lipoma	Nil	1
32	9 F	Suspected acute biliary pancreatitis	Nil	US, MRI	GA	Normal	Precluded need for ERCP	1
33	4 F	Cystic pancreatic mass on US	Nil	MRI	GA	Pancreatic pseudocyst	Surgery in urgency for traumatic rupture	1
34	18 F	Suspected CBDs	Nil	US	DP	Gallstones	Precluded need for ERCP; laparoscopic cholecystectomy	1
35	18 F	Perigastric abscess at US	PEG, holoprosencephaly	EGD, US	GA	Perigastric abscess	Surgical drainage	0
36	18 M	Recurrent hypoglycemia	Nil	MRI	DP	Solid hypoechogenic hypervascular lesion of uncinate process	FNA with 25 G (diagnosis of insulinomas); Medical therapy	2
37	18 F	Suspected acute biliary pancreatitis	Nil	US, CT	DP	Gallstones, edematous pancreatitis	Precluded need for ERCP; laparoscopic cholecystectomy	1
38	16 M	Suspected CBDs	Nil	US	DP	Gallstones and CBDs	Stones extracted at ERCP	2
39	3 F	Encopresis	Sacrococcygeal Yolk Sac Tumor	MRI	DP	Pararectal lesion	Surgical intervention (recurrent disease)	2
40	13 M	Suspected Crohn’s anal disease	Ilelonic Crohn’s disease	Colonoscopy, MRI	NS	Perianal abscess	Surgical intervention	2

*CBD* indicates common bile duct; *CBDs* indicates common bile duct stones; *CT* indicates computerized tomography; *DP* indicates deep sedation; *EGD* indicates Esophagogastroduodenoscopy; *ERCP* indicates endoscopic retrograde cholangiopancreatography; *EUS* indicates endoscopic ultrasound; *F* indicates female; *FNA* indicates fine needle aspiration; *GA* indicates general anesthesia; *M* indicates male; *MRI* indicates magnetic resonance imaging; *NET* indicates neuroendocrine tumor; *NS* indicates non sedation; *PEG* indicates percutaneous endoscopic gastrostomy; *PET* with Ga-DOTATOC indicates Gallium-68-somatostatin receptor positron emission tomography; *US* indicates ultrasound

### Anesthesia

For upper EUS, deep sedation with propofol was used in 22 (46.8%) procedures, whereas general anesthesia with endotracheal intubation was performed in 10 (21.3%) procedures. For lower EUS, 14 (29.8%) procedures were managed without sedation and only one procedure (2.1%) was approached with deep sedation due to the very young age of the patient and the presence of comorbidity (case n. 39). No sedation- or anesthesia-related complications occurred.

### Pancreatobiliary system

The pancreatobiliary system was endosonographically evaluated in 28 (59.6%) procedures, including 3(6.4%) EUS-FNA. The indications for EUS were: suspected CBDs (*n* = 8, 28.6%), suspected acute biliary pancreatitis (*n* = 7, 25%), recurrent/chronic pancreatitis (*n* = 4, 14.3%), suspected CBDs in patients with ulcerative colitis (*n* = 3, 10.7%), cystic pancreatic mass (n = 3, 10.7%), recurrent hypoglycemia (*n* = 2, 7.1%), and drainage of pseudocyst (*n* = 1, 3.6%). EUS for suspected CBDs was performed in the presence of cholestatic liver biochemistry with imaging suggestive of gallstones by US and MRI. Out of the 8 cases performed, 2 patients’ EUS showed the presence of CBDs, which were retrieved by ERCP during the same sedation session.

In the 7 cases of clinically and radiologically suspected acute biliary pancreatitis, EUS showed normal pancreatic parenchyma in 2/7 (28.6%) patients; endosonographic criteria for acute edematous pancreatitis with gallstones without CBDs in 4/7 (57.1%) patients; and acute necrotizing pancreatitis with gallstones without CBDs in one patient (14.3%). After 6 weeks, this latter patient (case n. 21) developed a voluminous pseudocyst with recurrent abdominal pain. Transgastric drainage was performed and a metallic stent was implanted. After an additional 6 weeks, CT imaging confirmed the cyst resolution and the stent was removed endoscopically.

In the 3 cases of recurrent pancreatitis (case n. 5, 14, 28), EUS showed endosonographic criteria for chronic pancreatitis without requiring further interventions. One of these patients presented with another episode of acute pancreatitis one year later. EUS was performed and showed the same results.

In 3 patients affected by ulcerative colitis with the presence of cholestatic liver biochemistry (case n. 3, 23, 25), MRI showed intrahepatic sclerosing cholangitis and CBDs were suspected. At the EUS examination, no stones were revealed and no ERCP was performed.

**Fig. 1:**
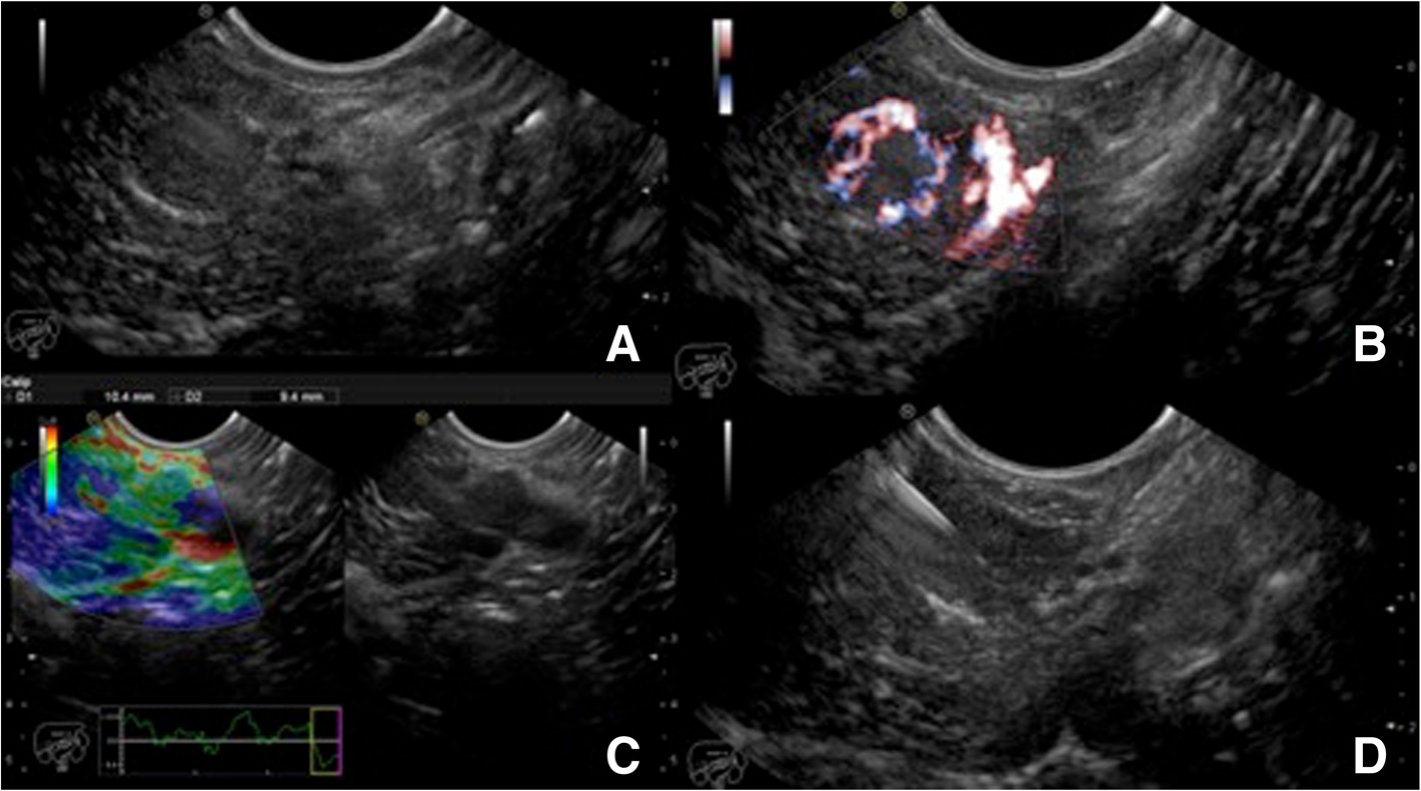
**a**: Endoscopic ultrasound (EUS) detection of solid hypoechogenic lesion with distinct boundaries in the tail of the pancreas. **b**: Color Doppler application revealing a hypervascular lesion. **c**: Elastography application revealing lower elasticity values compared to healthy pancreas. **d**: EUS-guided Fine Needle Aspiration (FNA) with a 25 G needle, yielding the final diagnosis of insulinomas

**Fig. 2:**
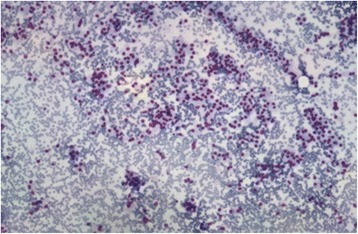
Fine needle aspirate showed single dispersed, uniform neoplastic cells, which rarely collect in clusters. The neoplastic cells appear round to oval and bland with eccentrically located nuclei (plasmacytoid appearance). No mitosis and no necrosis are observed in the background. Hematoxylin-Eosin 4× magnification

In the 2 patients with recurring episodes of hypoglycemia, EUS detected a solid hypoechogenic, hypervascular lesion with distinct boundaries of the uncinate process in one patient (case n. 36) and of the tail in the other patient (case n. 8), with lower elasticity values compared to a healthy pancreas. EUS-FNA was performed with a 25 G needle and a diagnosis of insulinoma was made in both cases (Fig. 1a-d, Fig. 2). Medical therapy was started in the first patient due to the advanced disease, whereas surgical resection was planned for the second patient.

The 2 patients with cystic pancreatic masses on US were referred to our center for EUS (cases n. 16, 33). In one case, EUS-FNA was performed. The endosonographic characteristics and pancreatic cyst fluid analysis were suggestive of a voluminous serous cystadenoma of the pancreatic head. EUS was repeated after 1 year due to acute pancreatitis, which demonstrated an increase in the cyst size with compression of the common bile and Wirsung ducts. Consequently, the patient underwent successful Whipple’s resection. In the second case, EUS diagnosed a pancreatic pseudocyst. A linear ultrasound bronchoscope was used only in this child (case n. 33) due to the very young age of the patient. Endoscopic drainage was planned but not performed because an emergency surgery was required for the rupture of the pseudocyst due to an abdominal trauma. The postoperative period was uneventful.

Among patients who underwent EUS for suspected CBDs or biliary pancreatitis, 12 of them (cases 1, 4, 6, 7, 10, 12, 15, 21, 26, 29, 34, 37; Table 2) avoided ERCP and underwent laparoscopic elective cholecystectomy, with a 4-week surgical follow-up. Five other cases (cases 2, 3, 23, 25, 32; Table 2) avoided ERCP, but those presenting with comorbidities affecting the biliary duct (e.g., sclerosing cholangitis, cases 3, 23, 25) were followed-up by abdominal ultrasound and/or MRI and biology tests according to the ACG guidelines 2015; the two patients who underwent EUS for pancreatitis in absence of other pancreatobiliary comorbidities were followed-up clinically and with biology tests at 6 and 12 months, including complete hepatic function tests, CRP and lipase, documenting a complete normalization of both clinic and biology.

### Upper GI tract

The indications for upper GI tract EUS included: characterization of duodenal polyp, gastric submucosal lesion, and perigastric abscess.

In one patient (case n. 20), bioptic specimens were suspicious for a neuroendocrine tumor (NET) of the posterior wall of the duodenal bulb. A duodenal hypoechoic, round-shaped, hypervascular lesion that originated in the submucosa and infiltrated the muscularis propria was detected. Surgical resection was required. Histology confirmed the diagnosis of NET G2, according to the 2010 World Health Organization classification [18]. Follow-up was scheduled every 6 months; CT, Gallium-68-somatostatin receptor positron emission tomography (PET with Ga-DOTATOC), EUS and plasmatic chromogranin A levels were all negative.

The second patient (case n. 31) who received upper GI EUS was referred for an endosonographic evaluation of a gastric subepithelial lesion. EUS with contrast enhanced showed a hyperechogenic submucosal lesion with regular margins suggestive of a lipoma was observed. The aspect of the mucosa was normal.

The third patient (case n. 35) presented with holoprosencephaly and a percutaneous endoscopic gastrostomy (PEG). The patient developed a peristomal infection with a perigastric abscess. EUS was performed to characterize and drain the lesion, which was not possible due to its location and surgery was required.

### Lower GI tract

Fifteen lower EUS procedures (31.9%) were performed. Nine children had suspected anal Crohn’s disease. Normal endosonographic findings were found in 3/9 cases. Three children (cases n. 24, 27, 30) had abscesses with extra sphincteric fistulas, whereas 1 patient had a perianal abscess (case n. 40). These four patients were treated surgically. In 1 child (case n. 11), a trans-sphincteric fistula was observed and medical therapy was started. EUS was performed bi-yearly to evaluate the response to therapy. Six months after the beginning of therapy, residual inflammation was demonstrated, but at 1 year a complete resolution was obtained.

In 1 child (case n. 19), EUS showed an extra-sphincteric fistula and medical therapy was started. Six months later, EUS demonstrated a complete resolution. In 2 children (cases n. 17, 22) the indication for EUS was fecal incontinence after surgery for Hirschsprung disease. EUS showed an interruption of the internal anal sphincter.

The last case (case n. 39) was a child with encopresis and previous surgery for sacrococcygeal yolk sac tumor. EUS showed a pararectal lesion suspicious for recurrent disease. The patient underwent surgery and a histological examination confirmed the diagnosis.

### Clinical impact of EUS

According to the predefined criteria [16], 6 (12.8%) EUS procedures yielded no further information compared to previous imaging results (classified as score 0). Twenty-four (51%) procedures were classified as score 1 because EUS established a definitive diagnosis or excluded a suspected pathological condition, thereby avoiding more invasive procedures. In the remaining 16 (34.1%) cases, EUS showed specific findings that allowed for targeted therapy (classified as score 2).

In one case (2.1%) EUS yielded significant results and allowed endoscopic therapy with EUS-guided cyst-gastrostome placement (classified as score 3). Overall, EUS had a positive clinical impact (score 1 + 2 + 3) in 41 (87.2%) procedures, affecting the subsequent clinical management.

According to the EUS findings, the therapeutic management was established as: medical therapy in the 5 patients affected by Crohn’s disease and in one patient with a neuroendocrine tumor; surgical intervention in 8 patients; and endoscopic therapy in 3 patients.

### Pediatric EUS and EUS-FNA cases in the literature

Table 3 shows the most relevant studies in the literature evaluating the application of EUS and EUS-FNA in pediatric populations. From 1998 to 2016, 10 studies [1, 3, 8, 11–17] were published with a total of 413 patients and 456 EUS (of which 69 (15.1%) were EUS-FNA) evaluated. Five studies were performed in the USA [11–13, 15, 17], 3 in Europe [1, 14, 16] and 2 in Asia [3, 8]. The main indication for EUS was the investigation of the pancreatobiliary tract in 324 (71.1%) cases. EUS-related complications were reported in only 3 studies [1, 8, 11], with an incidence rate ranging between 1.96% and 3.8%. Only 7/10 studies [1, 3, 8, 11, 12, 15, 16] evaluated the clinical impact of EUS, and these reported a positive impact in an average of 73.5% (range 35.5–98%) of cases.

**Table 3:** Summary of the current relevant literature and comparison with the present results

					Indications no, (%)						
Study	No. patients	No. EUS	Time frame (No. years)	Age (y), range (mean)	Pancreatobiliary	Rectum	Stomach	Esophagus	Duodenum	Other	EUS-FNA no, (%)
Roseau et al. 1998 [14]	18	23	7	4–16 (12)	8(34.8)	6(26.1)	6(26.1)	1(4.3)	1(4.3)	1(4.3)	0
Varadarajulu et al. 2005 [15]	14	15	3	5–17 (13)	15 (100)	0	0	0	0	0	3 (20)
Cohen et al. 2008 [3]	32	32	6	1.5–18 (12)	19 (59.4)	2 (6.3)	2 (6.3)	8 (25)	1 (3.1)	0	7 (21.9)
Bjerring et al. 2008 [16]	18	18	16	0.5–15 (12)	11 (61.1)	0	3 (16.7)	0	0	4 (22.2)	0
Attila et al. 2009 [13]	38	40	7	3–17 (13.5)	25 (62.5)	1 (2.5)	6 (15)	1 (2.5)	0	7 (17.5)	12 (30)
Al-Rashdan et al. 2010 [12]	56	58	8	4–18 (16)	42 (72.4)	4 (6.9)	1 (1.7)	1 (1.7)	0	10 (17.2)	15 (25.9)
Rosen et al. 2010 [17]	25	42	5	NA (14)	0	42 (100)	0	0	0	0	0
Scheers et al. 2015 [1]	48	52	14	2–17 (12)	52 (100)	0	0	0	0	0	12 (23.1)
Gordon et al. 2015 [11]	43	51	6	4–18 (14.5)	34 (66.7)	1 (1.9)	6 (11.8)	0	0	10 (19.6)	13 (25.5)
Mahajan et al. 2016 [8]	121	125	8	3–18 (15.2)	118 (94.4)	0	2 (1.6)	0	0	5 (4)	7 (5.6)
TOTAL (sum or weighted mean)	413	456	8	0.5–18 (14)	324 (71.1)	56 (12.3)	26 (5.7)	11 (2.4)	2 (0.4)	37 (8.1)	69 (15.1)
Present study	40	47	6	3–18 (15.1)	28 (59.6)	15 (31.9)	2 (4.3)	0	2 (4.3)	0	3 (6.4)

*NA* indicates not available; *EUS* indicates endoscopic ultrasound; *FNA* indicates fine needle aspiration

## Discussion

The present study illustrates the experience of a single high-volume endoscopic center in the application of EUS and EUS-FNA for several pediatric pancreatobiliary and GI pathologies. The case series included 47 procedures that were all technically successful, uneventful, and helpful for the clinical management of the patients, supporting the feasibility, safety and validity of EUS in children.

EUS techniques in pediatric still find limited indications, since other validated diagnostic modalities, such as US, CT, MRI or MRCP are more often preferred [14]*.* However, there is growing evidence (Table 3) to support the role and clinical impact of EUS, particularly to avoid unnecessary ERCP.

In the present study, as in the current literature, the most frequent indication for EUS was the investigation of the pancreatobiliary tract, in particular for suspected CBDs, acute/chronic pancreatitis, and pancreatobiliary abnormality [1, 3, 8, 12–16]. EUS, MRCP, and ERCP are the main diagnostic techniques for pancreatobiliary diseases [6]*.* For many years, ERCP has been considered the best preoperative diagnostic tool for the examination of the bile duct, although the related complication rate ranges from 5% to 10% in adults [4, 9, 19] and 3.4% to 28.5% in children [7]. Regarding the role of endoscopy in the management of suspected choledocholithiasis, the most recent American Society for Gastrointestinal Endoscopy (ASGE) guidelines indicate that clinicians should always perform a non-invasive test, such as EUS or MRCP, before ERCP [4, 6, 9, 19]*.* Indeed, two systematic reviews showed that MRCP has a high sensitivity (85% to 92%) and specificity (93% to 97%) for choledocholithiasis detection [20, 21]. However, EUS has been reported to be the most sensitive and highly specific diagnostic tool for choledocholithiasis and microlithiasis, which are responsible for at least half of all cases of acute pancreatitis. EUS was also found to be more accurate in evaluating microlithiasis of the gallbladder and early chronic/idiopathic pancreatic diseases [1, 3, 6, 22–28]. In our series, 18 cases presented with suspected biliary stones or acute biliary pancreatitis. EUS revealed CBDs in 2/18 children, who underwent ERCP during the same session. Thus, the EUS approach was helpful to avoid unnecessary ERCP and its associated risks in 16 (88.9%) patients with imaging suggestive for CBDs.

The therapeutic role of EUS has been clearly demonstrated in the management of pancreatic diseases. Commonly reported indications in children for EUS-FNA are the drainage of pancreatic collections, which is highly helpful in providing a definitive diagnosis [1, 29]*.* In the present study, EUS-FNA was performed in 3 patients and allowed a definitive diagnosis in all patients (2 pancreatic masses, 1 pancreatic cyst), who were then addressed to appropriate treatment. In the case of a cystic lesion, the cytopathological examination combined with the dosage of tumoral markers permitted a final diagnosis of serous cystadenoma. Traditionally, pancreatic pseudocysts were drained surgically or percutaneously (US or CT guided) [29, 30], but endoscopic drainage became the primary therapeutic modality in the mid-1980s [31]. Moreover, over the last decade, the role of EUS-guided pseudocyst drainage has dramatically increased due to its minimal invasiveness, lower costs, and lower complication rates [1, 32–36]. In the present case series, the child presenting with a pseudocyst and persistent abdominal pain following acute pancreatitis underwent a successful EUS-guided drainage.

EUS is also a relevant tool in the management of GI pathologies. Indeed, the ability of EUS to differentiate GI wall layers and identify extra-luminal structures makes it the best technique to study mucosal/submucosal lesions observed during conventional endoscopy [12, 29]*.* In the present study, EUS allowed the precise definition of the invasion of the muscularis layer in a patient with duodenal NET, preventing a non-radical endoscopic resection in favor of an adequate surgical treatment.

Regarding the application in the lower GI tract, EUS plays a major role in rectal cancer staging in the adult population [37, 38]. In children, EUS has been mainly used to evaluate anorectal anomalies, anal sphincter defects, and anal Crohn’s disease [3, 39]. In the present series, as in the previous literature [17, 40], EUS examination was found to be very precise in describing anorectal normal and abnormal anatomy, which guided the subsequent medical/surgical management. EUS was also useful in the follow-up period to evaluate the response to Crohn’s disease therapy. It must be noted, however, that the most common imaging modalities for the evaluation of anorectal anatomy remain CT and pelvic MRI. Both of these techniques have drawbacks: CT is associated with radiation exposure while MRI application is limited by high costs and restricted access in many centers [39, 41]. Moreover, in very young children, these methods require sedation. Conversely, EUS has the advantage that it may be performed at the same time as colonoscopy by a gastroenterologist, who can interpret both the clinical and imagery observations simultaneously and perform ERCP during the same session, if needed [17]. However, the final choice of which imaging modality to apply currently remains mainly dependent on institutional resources and clinical expertise.

The present study has some limitations. First, it is a retrospective analysis of data from a single high-volume center. The sample size is relatively small, with younger children and infants not adequately represented; indeed, the majority of the patients treated and evaluated were adolescents, limiting the possibility to generalize results to other ages. Finally, the paucity of EUS-FNA procedures performed does not allow the drawing of definitive conclusions.

Currently, the use of EUS in children is limited by the low availability of echoendoscopes in most pediatric centers together with the scarce experience and training of most pediatric gastroenterologists. In the near future, it is advisable that pediatric gastroenterologists acquire a specific expertise with EUS to extend the use of this diagnostic and therapeutic technique in pediatric populations.

## Conclusion

This single center case series supports the applicability, feasibility, and safety of EUS and EUS-FNA in the management of pediatric pancreatobiliary and GI disorders. Further research and large-scale studies are needed to standardize the indications and applications for EUS in pediatric populations.

## Abbreviations

ASA: American Society of Anesthesiologists
CBDs: Common bile duct stones
CT: Computed tomography
ERCP: Endoscopic retrograde cholangiopancreatography
EUS: Endoscopic ultrasound
FNA: Fine needle aspiration
GI: Gastrointestinal
MRCP: Magnetic resonance cholangiopancreatography
MRI: Magnetic resonance imaging
NET: Neuroendocrine tumor
US: Ultrasound
